# Leveraging Telemedicine for Chronic Disease Management in Low- and Middle-Income Countries During Covid-19

**DOI:** 10.5334/gh.852

**Published:** 2020-09-15

**Authors:** Michael A. Hoffer-Hawlik, Andrew E. Moran, Daniel Burka, Prabhdeep Kaur, Jun Cai, Thomas R. Frieden, Reena Gupta

**Affiliations:** 1 Columbia University Vagelos College of Physicians and Surgeons, New York, NY, US; 2 Resolve to Save Lives, New York, NY, US; 3 National Institute of Epidemiology, Indian Council of Medical Research, Chennai, Tamil Nadu, IN; 4 State Key Laboratory of Cardiovascular Disease, Hypertension Center, Fu Wai Hospital, National Center for Cardiovascular Diseases, Peking Union Medical College and Chinese Academy of Medical Sciences, Beijing, CN; 5 Division of General Internal Medicine, Department of Medicine, University of California San Francisco, US

**Keywords:** telemedicine, Covid-19, global health, chronic diseases

## Abstract

In response to the Covid-19 pandemic, many low- and middle-income countries (LMICs) expanded access to telemedicine to maintain essential health services. Although there has been attention to the accelerated growth of telemedicine in the United States and other high-income countries, the telemedicine revolution may have an even greater benefit in LMICs, where it could improve health care access for vulnerable and geographically remote patients. In this article, we survey the expansion of telemedicine for chronic disease management in LMICs and describe seven key steps needed to implement telemedicine in LMIC settings. Telemedicine can not only maintain essential medical care for chronic disease patients in LMICs throughout the Covid-19 pandemic, but also strengthen primary health care delivery and reduce socio-economic disparities in health care access over the long-term.

In response to the Covid-19 pandemic, many low- and middle-income countries (LMICs) expanded access to telemedicine to maintain essential medical care when face-to-face visits are unsafe. Although there has been attention to the accelerated growth of telemedicine services in the United States and other high-income countries, the telemedicine revolution may have an even greater benefit in LMICs, where it could improve health care access for vulnerable and geographically remote patients [1,2].

LMIC patients with chronic noncommunicable diseases such as hypertension, diabetes, or chronic obstructive lung disease have increased risk of severe Covid-19 [3]. Before the pandemic, patients with chronic diseases living in rural regions had to travel long distances, at considerable out-of-pocket cost, to reach the nearest health care facility. During the Covid-19 pandemic, in-person clinical care is often limited by government-imposed travel restrictions, strains on facility infection control measures, and advice to people with chronic illness to reduce contacts with others. Postponed outpatient clinic visits and prescription renewals may exacerbate chronic diseases, accelerate acute complications, further increase risk of severe Covid-19 illness, and thereby increase the stress on already-overwhelmed hospitals [4].

New telemedicine-promoting policies and ubiquitous mobile phone access in many LMICs now raise the possibility that telemedicine could help bridge gaps in care for chronic medical conditions. Even after Covid-19 is controlled, telemedicine has the potential to address persistent obstacles to primary health care in LMICs, including scarcity of trained health care workers, difficulty of patient transportation, and in-person care costs. As LMICs implement or broaden telemedicine services, a systematic approach will ensure safe and equitable access to essential chronic disease care both during and after the Covid-19 pandemic. Seven key components are required for LMIC health systems to adopt telemedicine (Figure 1) [5].

**Figure 1:**
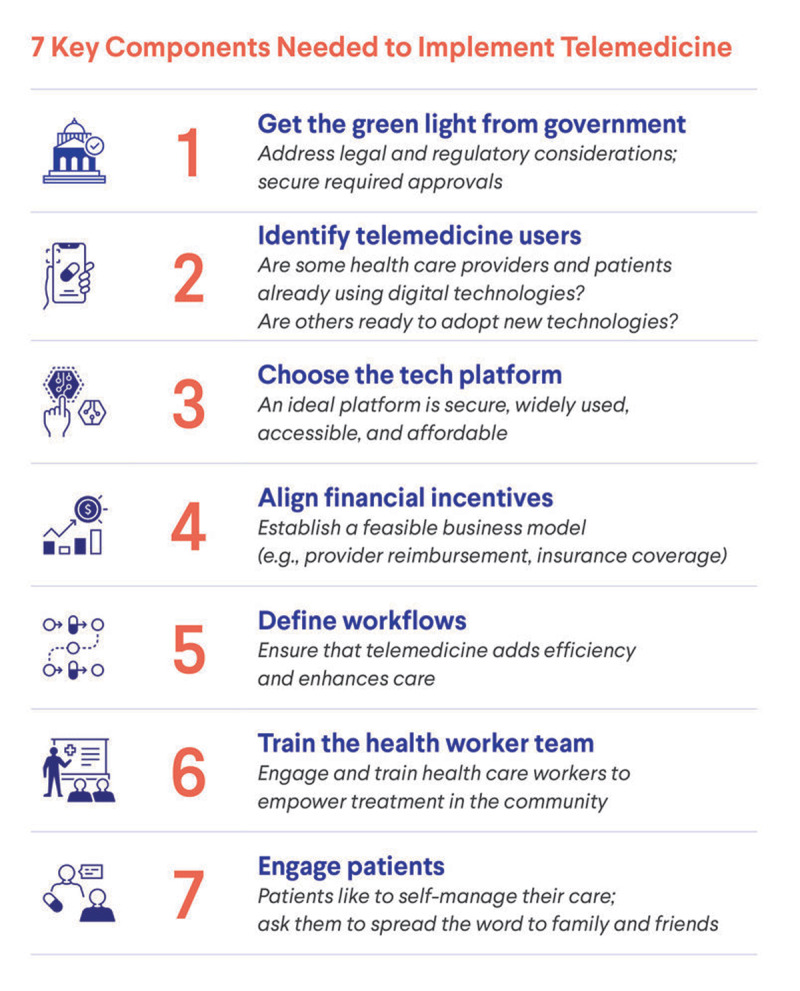
Seven key components needed to implement telemedicine in low- and middle-income countries [5].

## Legal and regulatory considerations

Until recently, clear government regulations to both enable and provide the boundaries of telemedicine services did not exist in many LMICs [6]. As Covid-19 spread, many governments expanded regulatory permissions rapidly, and some incorporated reimbursement policies to facilitate conversion of in-person visits to telemedicine consultations. In February 2020, the Chinese central government released guidelines that increased reimbursement coverage for follow-up online medical consultations and promoted doorstep delivery of prescriptions acquired through ‘internet hospitals’ [7]. India’s Ministry of Health and Family Welfare released national telemedicine practice guidelines in March 2020 [8]. This framework provided instructions for prescribing medications and conducting follow-up care for chronic diseases, thereby enabling patients to consult from home with medical providers via telemedicine services.

## Identifying telemedicine providers

Conversion of scheduled office appointments to telemedicine visits with existing providers has maintained continuity of care for many patients while limiting both patient and clinician travel exposure. Leveraging currently employed clinicians to conduct teleconsults allows practices to use existing human resources, referral networks, and provider workflows. An alternative approach, exemplified by the Indonesian health platform Halodoc, is to hire or contract with a telemedicine-dedicated pool of clinicians who provide consultations for health care workers in the community or patients at home [9]. Implementing telehealth call centers provides flexible staffing capacity, leading to more real-time visits and faster response times. Health care systems which use electronic health records are well suited to telemedicine, as clinicians can access patient files remotely. Despite the workforce efficiencies of telehealth call centers, extensive investment is required to provide management oversight and to create new clinical protocols and safety monitoring procedures.

## Choosing telemedicine technology platforms

In the short-term, low-cost and widely-used communication platforms such as WhatsApp or WeChat are a means to rapidly expand telemedicine services in LMICs. Alternatively, dedicated mobile telemedicine applications allow patients to quickly and easily schedule appointments and communicate in real time with providers via video and audio capabilities on their personal devices. China’s Fu Wai Hospital ‘Hypertension Doctor’ smartphone app, which can be downloaded on iOS or Android mobile operating systems, provides two-way communication between patients and physicians for home blood pressure measurements, education, medication dose titration, and prescription refills [10].

## Aligning financial incentives

For resource-limited countries implementing new or adapting current telemedicine programs, it is essential to demonstrate long-term financial value to health care payors, health systems, physicians, and patients [11]. LMIC health care systems that operate under global budget or capitation models may find more seamless integration of low-cost telemedicine initiatives compared to systems with predominantly fee-for-service health insurance models. Where telemedicine consultations result in a fee, physicians should state expected costs with patients at the onset of the encounter to align patients’ health care goals and financial expectations.

## Designing telemedicine workflows

**Figure 2:**
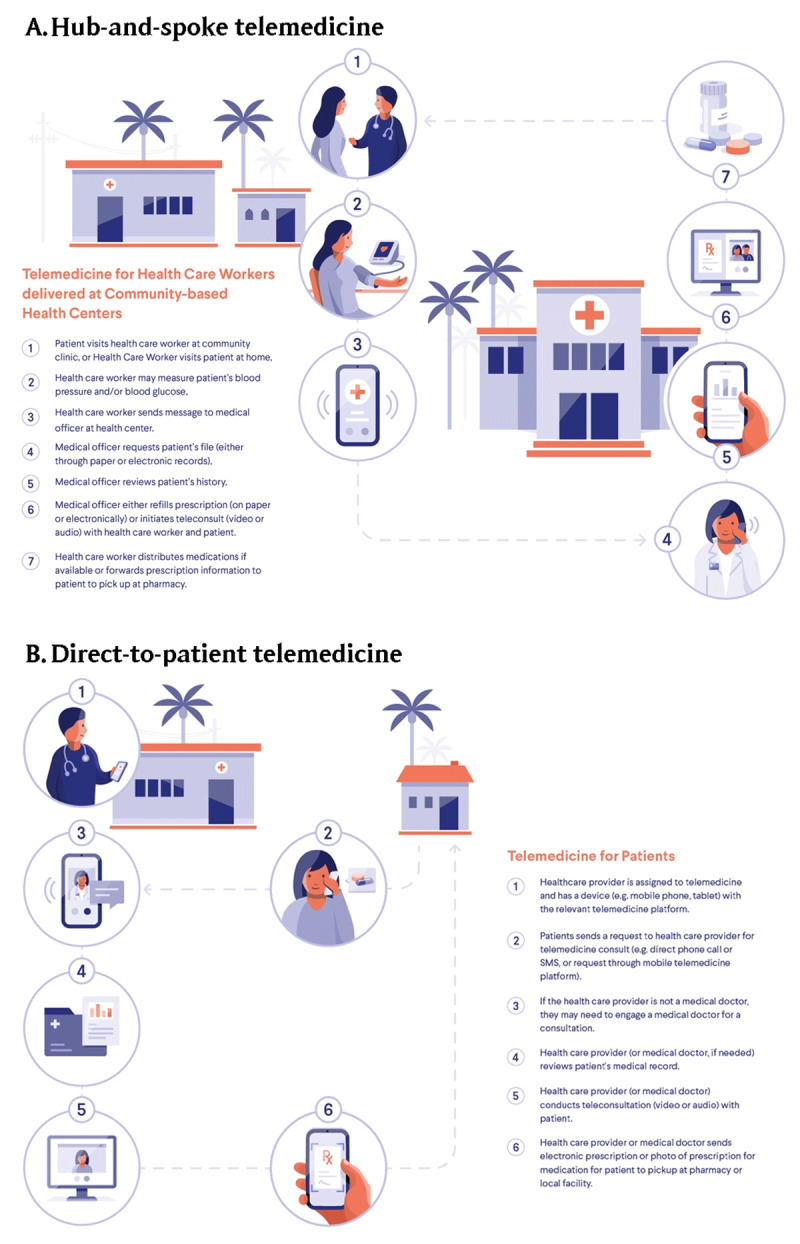
Telemedicine workflows for low- and middle-income countries. Hub-and-spoke models coordinate care between a central facility and community site (Panel A); direct-to-patient models coordinate care directly between health care provider and patient (Panel B).

In LMICs, practices must consider existing health care provider roles, practice functions, reimbursement, and regulatory compliance when designing telemedicine workflows. In the Philippines, the Taguig City government designed a telemedicine system around previous patient triage protocols for both suspected Covid-19 and non-Covid-19 chronic disease patients. After a telephone interview by representatives at the centralized City Health Office, patients are referred to the appropriate health care provider and offered contactless prescription deliveries [12]. Despite the advantages of telemedicine for treating chronic, non-urgent medical problems, there must be a clear pathway to referral for in-person care when a face-to-face physical examination or procedure is indicated. The Resolve to Save Lives global hypertension control initiative recently developed two telemedicine workflows—‘hub and spoke’ and ‘direct to patient’—to both maintain continuity of care and address emergency situations for established hypertension patients (Figure 2) [5].

## Health worker training and patient engagement

Engaging health care providers and patients is critical to creating a sustainable telemedicine program. Clear and simple ‘playbook’ training instructions facilitate rapid adoption by medical staff. Integrating a monitoring and evaluation framework will recognize care gaps and drive continuous quality improvement. Digital literacy training, access to interpreters or clinicians who speak local languages, and active outreach to high-risk individuals are cornerstones of patient-centered care that should be employed to promote telemedicine uptake and limit disparities among vulnerable populations. Encouraging LMIC patient utilization of telemedicine services through text messaging or online marketing efforts will limit spread of infection during the Covid-19 pandemic and lay the groundwork for ongoing health care improvement.

Telemedicine can play a key role not only in maintaining essential health services for chronic disease patients in LMICs during the Covid-19 pandemic, but also in long-term primary health care strengthening. As telemedicine spreads in LMICs, it will be important to monitor its impact on quality of care and address socio-economic disparities in telemedicine adoption. Covid-19 has catalyzed rapid expansion of telemedicine, but its longevity will hinge not on technology alone, but on government regulations, payment policies, and health system redesign. With concerted efforts, expansion of telemedicine can sustain and extend the reach of primary health care in LMICs during Covid-19 and beyond.
